# Identifying Change Fatigue in Nurses From Southwestern China via Latent Profile Analysis: A Multicenter Cross‐Sectional Study

**DOI:** 10.1155/jonm/7461404

**Published:** 2026-06-27

**Authors:** Lin Wang, Daoyuan Chai, Mingyue Wu, Yi Tang, Jie Mi

**Affiliations:** ^1^ Department of Anesthesiology, The First Affiliated Hospital of Chongqing Medical University, Chongqing, China, cqmu.edu.cn; ^2^ Department of Critical Care Medicine, The First Affiliated Hospital of Chongqing Medical University, Chongqing, China, cqmu.edu.cn

**Keywords:** change fatigue, emotional labor, latent profile analysis, nurses, nursing leadership, occupational health, psychological resilience, southwestern China, workforce management

## Abstract

**Objective:**

To identify latent profiles of change fatigue among nurses in Southwestern China and explore factors associated with distinct profiles.

**Background:**

Healthcare resources in Southwestern China are unevenly distributed, and the region features considerable ethnic diversity. Against the backdrop of ongoing reforms in the healthcare system, nurses—as frontline implementers—are constantly exposed to intensive and frequent updates in policies, technologies, and workflows, making them susceptible to change fatigue. This sustained exposure precipitates “change fatigue,” a syndrome that erodes psychological resilience and professional identity, and is prospectively linked to heightened turnover intention, measurable deterioration in nursing quality and an increased incidence of patient‐safety events.

**Methods:**

From July to September 2025, we recruited nurses from various tiers and types of medical institutions across Southwestern China. Data were collected using a general information questionnaire, the Change‐Related Stress Scale, the Grandey Emotional Labor Strategy Scale, the Connor‐Davidson Resilience Scale (CD‐RISC), and the Chinese Nurse Job Stressors Scale. Latent profile analysis (LPA) was employed to identify subgroups of change fatigue. Univariate analysis and multinomial logistic regression with Firth’s penalized likelihood estimation were used to examine factors associated with profile membership.

**Results:**

A total of 1383 valid questionnaires were included. LPA revealed three distinct profiles of change fatigue: low fatigue (17.5%, *n* = 242), moderate fatigue (61.6%, *n* = 852), and high fatigue (20.9%, *n* = 289). Multinomial logistic regression showed that nurses in the high fatigue group were significantly more likely to work in the intensive care unit (OR = 2.31, 95% CI: 1.40–3.85, *p* = 0.001) and internal medicine (OR = 2.12, 95% CI: 1.26–3.61, *p* = 0.005). Working 1–2 night shifts per week increased odds of high fatigue by 53% (OR = 1.53, 95% CI: 1.03–2.27, *p* = 0.034). Compared with minimal emotional labor (Level 1, 14–30 points), both moderate (Level 2, 31–50 points: OR = 0.14, *p* = 0.030) and high (Level 3, 51–70 points: OR = 0.09, *p* = 0.010) emotional labor levels were associated with significantly lower odds of high fatigue, suggesting that deficient emotional labor engagement may represent a risk configuration. Work stress Level 3 increased odds of high fatigue 33‐fold (OR = 32.71, 95% CI: 13.27–103.37, *p* < 0.001). Psychological resilience showed no independent significant association with profile membership in multivariate models.

**Conclusion:**

Change fatigue exhibits a heterogeneous tripartite structure. Minimal emotional labor engagement (Level 1) was associated with higher odds of high fatigue compared with moderate and high levels, suggesting that deficient emotional labor may represent a distinct risk configuration. Both modifiable workplace factors (ICU/internal medicine placement, night shifts, and job stress) and emotional labor patterns were associated with profile membership, supporting the potential value of organizational interventions and targeted emotional labor training.

**Implications for Nursing Management:**

These findings provide an evidence‐based foundation for precision prevention. Nursing leaders should integrate the six‐item Change Fatigue Measurement Scale into routine occupational health surveillance to enable profile‐based risk stratification. High‐fatigue nurses (20.9%) require immediate workload relief and mental health referral; moderate‐fatigue nurses (61.6%) represent a critical prevention window for resilience training and peer support; low‐fatigue nurses (17.5%) should serve as peer mentors and change champions. Priority interventions should target ICU and internal medicine units given the 2‐ to 2.3‐fold increased high‐fatigue risk. Leaders should limit consecutive night duties to ≤ 2 shifts, ensure ≥ 11 h rest between shifts, and enforce weekly overtime caps (≤ 8 h). Differentiated emotional labor training is essential: Nurses with minimal engagement need professional identity strengthening and authentic expression workshops, while those with excessive engagement require boundary‐setting training and mindfulness‐based stress reduction. Resilience‐building interventions must be embedded within organizational support initiatives rather than implemented as standalone programs. In ethnically diverse, resource‐constrained contexts, culturally tailored change communications and phased implementation timelines are critical to avoid “reform stacking.”

## 1. Introduction

Change fatigue is defined as a complex psychological state comprising stress, exhaustion, and burnout that arises in response to persistent and rapid organizational changes, frequently accompanied by feelings of conflict and helplessness [1, 2]. Against the backdrop of a rapidly evolving global healthcare landscape, ongoing organizational transformations including technological innovation, restructuring of nursing resources, and reforms to performance systems have become the norm [3]. As pivotal actors in clinical care, nurses must not only maintain patient safety and care quality under high‐workload conditions but also continually acquire new skills and adapt to updated protocols. This dual responsibility renders them especially vulnerable to change‐related stress [4, 5, 6]. Previous cross‐sectional studies have shown that nurses experience significantly higher levels of change fatigue compared to other health professionals, which in turn predicts burnout, reduced job satisfaction, team disengagement, and increased turnover intention [3, 7, 8, 9]. More critically, change fatigue not only compromises nurses’ physical and mental well‐being but also erodes organizational commitment, impedes the implementation of change, and sustains a vicious cycle of “change‐‐fatigue‐‐resistance‐further change failure” [7, 10, 11].

In Southwestern China, which is marked by uneven distribution of healthcare resources and ethnic diversity, nurses are disproportionately exposed to frequent policy and technological shifts. Given that research on change fatigue among hospital nurses is still in its early stages, studies focusing on nurses in different tiers of hospitals in Southwest China remain extremely scarce. No research to date has stratified samples by hospital tier or explored contextual moderators such as ethnic diversity, mountainous geography, and tiered healthcare reforms. This evidence gap hinders accurate assessment of the regional prevalence of change fatigue and limits in‐depth understanding of regional characteristics and key influencing factors.

Latent profile analysis (LPA) is a probabilistic modeling technique that identifies latent subgroups within a population based on multidimensional continuous indicators, without requiring prior classification [12, 13, 14]. This approach allows for the establishment of a “typology‐prediction‐verification” closed loop, enabling researchers to move beyond variable‐centered approaches to identify distinct subgroups with shared characteristics [12, 13]. In this study, the application of LPA will help identify latent subgroups with similar change fatigue characteristic patterns, thereby providing a basis for implementing targeted intervention strategies [14].

In light of this, the present study aims to explore the latent profiles of change fatigue among nurses in Southwestern China and analyze their influencing factors through a multi‐center, large‐sample survey, with the goal of providing an evidence‐based foundation for developing precise nursing management strategies and policy recommendations.

## 2. Methods

### 2.1. Ethical Approval

This study was approved by the Ethics Committee of The First Affiliated Hospital of Chongqing Medical University (Ethical approval number ZZ2025‐480‐01; Ethics registration number: ChiCTR2500112164). To ensure fully informed and voluntary participation, an informed consent section was included at the beginning of the survey, detailing the nature, purpose, benefits, and significance of the research. Participant privacy was strictly protected through data anonymization and restriction of data usage to scientific research purposes only.

### 2.2. Study Design and Participants

A multicenter cross‐sectional survey design was adopted. Using convenience sampling, a total of 1448 nurses were recruited. The inclusion criteria were as follows: (a) registered nurse; (b) age ≥ 18 years; (c) absence of psychiatric or cognitive disorders; and (d) voluntary participation. Exclusion criteria included (a) nurses engaged in advanced studies at other hospitals or on leave during the survey period; (b) those pursuing a master’s degree or higher; and (c) nurses in internship or probationary periods. Nurses pursuing master’s degrees or higher were excluded because they typically hold dual roles involving both clinical practice and research/teaching responsibilities, which may confound the assessment of frontline change fatigue. The inclusion of head nurses and supervisory staff (7.81% of the sample) may introduce potential bias, as their leadership roles could influence their perception of organizational change. We attempted to minimize response bias by ensuring that all participation was voluntary and anonymous, with no direct supervision during survey completion. However, we acknowledge this as a limitation and have addressed it in the Limitations section.

### 2.3. Data Collection

Data collection was conducted between July and September 2025. All survey investigators were trained to ensure accurate and consistent interpretation of the concepts and criteria involved in the study. The questionnaire was developed using the professional online survey platform Wenjuanxing (https://www.wjx.cn/), which generated a quick response (QR) code for questionnaire access. After obtaining informed consent from the nursing administration departments of participating hospitals, the QR code was distributed to head nurses of relevant departments, who then shared it with staff nurses. Nurses could scan the code via WeChat to access the survey. The first page of the questionnaire provided clear instructions, and participants were required to provide informed consent before proceeding. Completing the questionnaire took approximately 15–20 min. To ensure data quality, all items were set as mandatory, and each participant could submit responses only once. Additionally, questionnaires completed in less than 10 min or more than 30 min, as well as those showing patterned responses across items, were excluded. Participation was voluntary, and nurses retained the right to withdraw at any time. Finally, all data were collected through the Wenjuanxing platform.

### 2.4. Measures

#### 2.4.1. General Information

This section collected demographic and work‐related characteristics, including age, gender, type of employment, total years of working, marital and parenthood status, department, number of night shifts per month, professional title, administrative position, education level, daily overtime hours, and frequency of having less than 11 h of rest between two shifts within a week.

#### 2.4.2. Change Fatigue

The six‐item Change Fatigue Measurement Scale (CFMS) was selected as the only validated tool within the literature to examine the primary outcome of change fatigue score (CFS). The scale has shown good reliability and internal consistency in larger sample populations, including recent use in the nursing population by Brown [2]. The scale was originally developed as part of a suite of measurements to explore the impact of multiple organizational changes on employee well‐being, organizational commitment, and turnover intentions (sample item includes “I am tired of all the changes in this organization”) [1]. The CFMS uses a 7‐point Likert scale ranging from 1 (*strongly disagree*) to 7 (*strongly agree*) to determine a total CFS. The Chinese version of the Change Fatigue Scale (CFS) had a Cronbach’s alpha coefficient of 0.918.

#### 2.4.3. Emotional Labor

Emotional labor was measured with the 14‐item Emotional Labor Scale (ELS) originally developed by Grandey [15] and subsequently translated and cross‐culturally adapted for Chinese nurses by Luo et al. [16]. The instrument comprises three subscales: surface acting (seven items), deep acting (three items), and emotional expression requirements (four items). All items are rated on a six‐point Likert scale (1 = *strongly disagree* to 6 = *strongly agree*); higher scores indicate more frequent use of the corresponding strategy. A composite score is calculated as the sum of all 14 items, with higher values reflecting greater overall emotional labor intensity. The Chinese ELS has demonstrated satisfactory psychometric properties in previous investigations [17]. In the present sample, Cronbach’s α was 0.860 for the total scale and 0.869, 0.891, and 0.754 for the surface acting, deep acting, and emotional expression requirements subscales, respectively.

#### 2.4.4. Resilience Scale

Resilience is a positive adaptive process in which an individual, when confronted with significant stress or traumatic events, dynamically integrates biological, psychological, and social resources to maintain or rapidly restore psychosomatic homeostasis. The Chinese version of the Connor‐Davidson Resilience Scale (CD‐RISC), translated and adapted by Yu and Zhang et al. [18], was used to assess psychological resilience. This scale consists of 25 items grouped into three dimensions: tenacity (13 items), strength (8 items), and optimism (4 items). Responses are rated on a 5‐point Likert scale ranging from 0 (“*not true at all*”) to 4 (“*true nearly all the time*”). The total score ranges from 0 to 100, with higher scores indicating greater resilience. The Chinese version of the scale demonstrated good internal consistency, with a Cronbach’s α of 0.91.

#### 2.4.5. Chinese Nurses’ Job Stressors Scale

Nurses’ work‐related stress was evaluated using the Chinese Nurses’ Job Stressors Scale, developed by Li Xiaomei and Liu Yanjun in 2000 and tailored to the Chinese context [19]. The scale comprises 35 items distributed across five subscales: nursing profession and work, workload and time allocation, work environment and resources, patient care, and management and interpersonal relationships. Items are scored on a 4‐point scale, with higher scores reflecting higher levels of stress. The scale showed excellent reliability, with a Cronbach’s α of 0.946.

### 2.5. Data Analysis

Data were analyzed using SPSS 25.0 (SPSS Inc., Chicago, IL, USA), Mplus 8.3, and R 4.3.1 (with the “logistf” package). The statistical significance threshold was set at *p* < 0.05. Continuous variables with normal distribution were expressed as mean ± standard deviation (SD), while non‐normally distributed variables were summarized as median (P25, P75). Group comparisons were performed using ANOVA or the Kruskal–Wallis H test, as appropriate. Categorical variables were described using frequencies and percentages.

LPA was conducted using scores from all six items of the Change Fatigue Scale as indicator variables. Models with one to five latent profiles were estimated sequentially using robust maximum likelihood estimation (MLR) with 1000 random starting values and 300 final‐stage optimizations to prevent convergence on local maxima. Model fit was evaluated using the Akaike information criterion (AIC), Bayesian information criterion (BIC), sample‐size adjusted BIC (aBIC), entropy, the bootstrap likelihood ratio test (BLRT), and the Lo–Mendell–Rubin adjusted likelihood ratio test (LMR). Lower values of AIC, BIC, and aBIC indicated better fit, while entropy values closer to 1 suggested more accurate classification. A significant result (*p* < 0.05) for BLRT or LMR indicated that the k‐profile model fits better than the (k‐1)‐profile model. The final model was selected based on statistical indices, profile sample sizes (minimum ≥ 5%), theoretical interpretability, and practical utility [12, 13, 20].

Categorization of continuous predictors:

For multinomial logistic regression analysis, continuous variables were categorized based on distributional characteristics and clinical relevance:

Emotional labor (range 14–84) is as follows: scores 14‐30 (low/Level 1), 31‐50 (moderate/Level 2), and 51‐70 (high/Level 3), corresponding to approximate tertiles with consideration of the scale’s theoretical range.

Psychological resilience (range 0–100) is as follows: scores 0‐50 (low), 51‐75 (moderate), and 76‐100 (high), based on CD‐RISC normative data and previous validation studies in Chinese nursing populations [18].

Job stress (range 35–140) is as follows: scores 35‐70 (low), 71‐105 (moderate), and 106‐140 (high), corresponding to quartile distributions with the upper quartile representing clinically significant stress levels [19].

#### 2.5.1. Multinomial Logistic Regression

Multinomial logistic regression was used to examine factors associated with profile membership, with the moderate fatigue group serving as the reference category.

Addressing complete separation: Preliminary analysis revealed quasicomplete separation in the emotional labor variable for the low fatigue group (no participants in the lowest emotional labor category [14‐30] were classified into the low fatigue profile). To address this issue, we applied Firth’s penalized likelihood logistic regression using the “logistf” package in R, which reduces bias in parameter estimates through Jeffreys’ prior penalty and provides finite, less biased estimates under separation conditions [21]. This approach has been demonstrated superior to traditional maximum likelihood estimation when dealing with small samples or sparse data structures in nursing and health services research [21]. Odds ratios (ORs) and 95% confidence intervals (CI) were calculated. The reliability of all scales was assessed using Cronbach’s α.

## 3. Results

### 3.1. Demographic Characteristics

A total of 46 medical institutions from Southwest China were enrolled, comprising 42 general hospitals and 4 specialized hospitals. Overall, 1448 individuals were invited to participate in the survey; 1383 valid questionnaires were ultimately included in the analysis, yielding an effective response rate of 95.51%. The majority of participants were female (92.41%). The largest age group was 31–40 years (50.76%), followed by 26–30 years (28.71%) and 41–50 years (11.14%). Most nurses (78.09%) were from Grade A tertiary hospitals. In terms of marital status, 70.86% were married. The primary professional titles reported were nurse practitioner (42.30%) and senior nurse (37.24%). More than half of the participants (52.42%) had over 10 years of work experience, with the largest subgroup (42.01%) having 10–20 years of experience. Regarding departmental distribution, 321 participants (23.19%) worked in internal medicine, 261 (18.86%) in operating rooms, and 250 (18.06%) in other departments (primarily paramedical technical units). There were 198 participants (14.31%) from surgical departments, 175 (12.65%) from emergency departments, and 178 (12.86%) from intensive care units (ICUs). The vast majority (91.18%) served as frontline nurses, while a smaller proportion (7.81%) held managerial positions such as head nurse or above. In terms of night shift frequency, 54.09% worked 1–2 night shifts per week, though 5.64% reported working four or more night shifts weekly. Over half of the participants (50.83%) worked more than one hour of overtime daily, with 15.11% working 2–5 h or more. Regarding sleep duration, 67.75% slept 6–8 h per day, while 28.71% slept less than 6 h, and a small fraction (1.01%) slept fewer than 4 h. In terms of employment type, 292 (21.11%) were permanent staff nurses, and 1091 (78.89%) were contract‐based nurses (Table 1).

**TABLE 1:** Baseline characteristics of the respondents (*N* = 1383).

Item	Option	Frequency	Percentage (%)
Gender	Male	105	7.592
	Female	1278	92.408

Age range	18–25 years old	96	6.941
	26–30 years old	397	28.706
	31–40 years old	702	50.759
	41–50 years old	154	11.135
	50–60 years old	33	2.386
	Above 60 years old	1	0.072

Hospital grade	Grade A, Class III	1080	78.091
	Grade B, Class III	199	14.389
	Grade A, Class II	95	6.869
	Others	9	0.651

Marital status	Unmarried	358	25.886
	Married	980	70.86
	Divorced	45	3.254

Professional title	Nurse	213	15.401
	Senior Nurse	585	42.299
	Nurse‐in‐Charge	515	37.238
	Associate Chief Nurse and above	70	5.061

Educational background	Secondary Technical School	5	0.362
	Associate Degree	150	10.846
	Bachelor’s Degree	1200	86.768
	Postgraduate and above	28	2.025

Work experience	Less than 1 year	30	2.169
	1–2 years	71	5.134
	2–5 years	241	17.426
	5–10 years	316	22.849
	10–20 years	581	42.01
	More than 20 years	144	10.412

Work department	Surgery	198	14.306
	Emergency Department	175	12.645
	Operating Room	261	18.858
	Intensive Care Unit	178	12.861
	Internal Medicine	321	23.194
	Other	250	18.064

Employment type	Permanent Position	292	21.114
	Contract‐Based Position	1091	78.886

Position	Nurse	1261	91.179
	Head Nurse or Above	108	7.809
	Other	14	1.012

Frequency of mid/night shifts per week	1‐2 times	748	54.085
	3‐4times	247	17.86
	4 times or more	78	5.64
	0 times	310	22.415

Daily overtime hours beyond standard 8‐hour workday	0–1 h	680	49.168
	Within 2 h	457	33.044
	2–5 h	209	15.112
	More than 5 h	37	2.675

Daily sleep duration	Less than 4 h	14	1.012
	4–6 h	383	27.693
	6–8 h	937	67.751
	8 h or more	49	3.543

### 3.2. Classification and Characteristics of Change Fatigue

The results identified three distinct profiles of change fatigue: low change fatigue (*n* = 242, 17.50%), moderate change fatigue (*n* = 852, 61.61%), and high change fatigue (*n* = 289, 20.90%).

A series of five latent profile models were estimated to identify subgroups of change fatigue among nurses in Southwestern China. Model fit indices are presented in Table 2. As the number of latent profiles increased, the AIC, BIC, and sample‐size aBIC values decreased consistently, indicating improved model fit. Starting from the two‐profile model, both the LMR and BLRT yielded *p* values of 0.000, suggesting that each additional profile significantly enhanced model fit up to the five‐profile solution.

**TABLE 2:** Indicators for latent profile analysis of nurses’ change fatigue (*N* = 1383).

Latent class model	AIC	BIC	aBIC	P (LMR)	P (BLRT)	Entropy value	Class proportions
1	30668.26	30731.05	30692.93				
2	27591.34	27690.74	27630.39	0.000	0.000	0.935	0.233/0.767
3	24530.05	24666.09	24583.5	0.000	0.000	0.95	0.179/0.211/0.61
4	23807.14	23979.79	23874.97	0.000	0.000	0.933	0.168/0.459/0.213/0.16
5	23198.47	23407.75	23280.69	0.000	0.000	0.936	0.066/0.11/0.443/0.221/0.161

**FIGURE 1:**
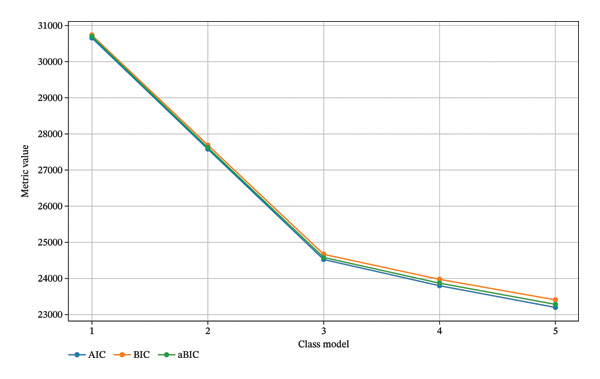
Scree plot of fit indices for latent profile models.

Model selection rationale: Although the five‐profile model achieved the lowest AIC (23,198.47), BIC (23,407.75), and aBIC (23,280.69), we selected the three‐profile solution as optimal based on comprehensive evaluation of statistical fit, classification quality, parsimony, and substantive interpretability [12, 13, 20]:

1. Statistical fit and classification quality: The three‐profile model demonstrated the highest entropy value (0.95) among all solutions, indicating superior classification accuracy and model stability. While the five‐profile model had comparable entropy (0.936), the four‐profile model showed decreased entropy (0.912), suggesting diminished class separation.
2. Parsimony and overfitting prevention: A scree plot illustrated the comparative fit of the five models across AIC, BIC, and aBIC (Figure 1). The most substantial improvements in fit were observed from the one‐ to three‐profile models. Beyond three profiles, the gains in fit were marginal (AIC reduction: 3 ⟶ 4 profiles: 892.47; 4 ⟶ 5 profiles: 412.31), and the risk of overfitting increased substantially [12, 20]. The LMR test, which compares k‐class versus (k‐1)‐class models, was significant for the 2‐class (*p* < 0.001) and 3‐class (*p* < 0.001) models but became nonsignificant for the 4‐class model (*p* = 0.082), indicating that additional profiles beyond three did not provide statistically meaningful improvement [12, 20].
3. Profile size and stability: In the four‐profile solution, the smallest profile comprised only 8.3% (*n* = 115) of the sample, and in the five‐profile solution, two profiles were smaller than 5% (4.2% and 3.8%), falling below the recommended threshold of 5%–10% for reliable latent class estimation [20]. Such small classes risk unstable parameter estimates and reduced generalizability.
4. Clinical interpretability and practical utility: The three‐profile solution yielded qualitatively distinct and clinically meaningful subgroups representing low, moderate, and high change fatigue states that align with existing theoretical frameworks and clinical observations [2, 13]. The four‐ and five‐profile solutions primarily fragmented the large “moderate fatigue” profile into subgroups differing quantitatively in severity rather than presenting qualitatively distinct patterns, offering no conceptually new insights and indicating statistical overfitting [13]. As a core tenet of person‐centered analysis is to identify substantively distinct subgroups rather than create arbitrary distinctions along a continuum, the three‐profile solution optimally balanced statistical fit with theoretical coherence [13].
5. Actionability for intervention: The tripartite classification supports straightforward clinical decision‐making: routine monitoring for the moderate group (61.6%), intensive intervention for the high‐fatigue group (20.9%), and maintenance of protective factors for the low‐fatigue group (17.5%) [13]. This aligns with precision prevention approaches that prioritize resource allocation to high‐risk subpopulations.

**FIGURE 2:**
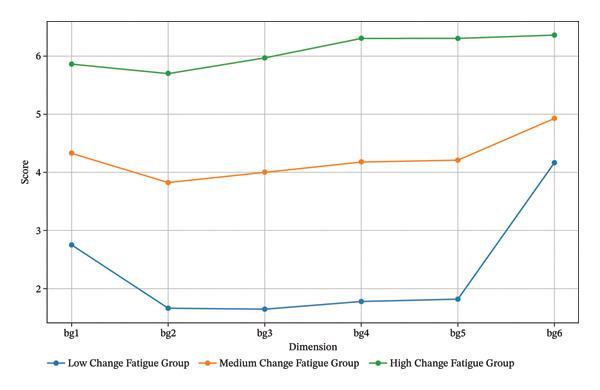
Mean scores of identified latent profiles in latent profile analysis.

The three nurse profiles identified through LPA demonstrated clear gradient differences across the six dimensions of change fatigue (bg1–bg6), supporting good discriminant validity (Figure 2). The high change fatigue profile scored highest across all dimensions, with mean values ranging from 5.7 to 6.4, reflecting the most intense experience of change‐related exhaustion—particularly on items bg4, bg5, and bg6. The moderate change fatigue profile displayed intermediate scores, with means between 3.8 and 4.9, suggesting a moderate level of adaptation coupled with perceptible strain. In contrast, the low change fatigue profile scored significantly lower than the other two groups across all dimensions, with scores as low as approximately 1.6 on items such as bg2 and bg3, indicating minimal change‐related fatigue and psychological burden. Accordingly, the three profiles were labeled as “High Change Fatigue,” “Moderate Change Fatigue,” and “Low Change Fatigue” (Figure 2).

### 3.3. Analysis of Factors Influencing Latent Profiles of Change Fatigue

No statistically significant differences were observed among the three profiles (low, moderate, and high fatigue) in demographic variables such as gender, age, marital status, education level, professional title, hospital grade, employment type, or administrative position (all *p* > 0.05). However, significant differences were detected in several work‐related and psychological variables (all *p* < 0.001).

The distribution of work departments differed significantly across the fatigue groups (*X* ^2^ = 21.25, *p* < 0.001). In the low fatigue group, the highest proportion worked in internal medicine (28.93%), followed by other departments (22.73%), while surgical and emergency departments accounted for lower proportions (13.64% and 6.20%, respectively). In the moderate fatigue group, internal medicine (23.94%) and other departments (19.60%) remained predominant, with emergency and surgical departments comprising 14.32% and 15.02%, respectively. In the high fatigue group, the proportions from internal medicine (25.6%) and ICUs (22.5%) increased markedly, whereas the shares from other departments and operating rooms decreased to 9.7% and 18.3%, respectively. Overall, increasing change fatigue was associated with a progressive redistribution of personnel toward high‐acuity units (e.g., ICU and internal medicine) and a relative reduction in miscellaneous departments.

Both night shift frequency (*X* ^2^ = 21.25, *p* = 0.002) and daily overtime hours (*X* ^2^ = 28.36, *p* < 0.001) exhibited a graded increase with fatigue level. The high fatigue group contained significantly more nurses working frequent night shifts and longer overtime. Sleep duration also differed significantly (*X* ^2^ = 46.66, *p* < 0.001), with a restorative pattern observed: 37.72% of the high fatigue group reported short sleep (4–6 h), whereas over 80% of the low fatigue group reported adequate sleep (≥ 6 h).

Emotional labor, psychological resilience, and job stress also differed markedly across profiles. Emotional labor level showed the most pronounced disparity (*X* ^2^ = 118.55, *p* < 0.001), with the high fatigue group containing more individuals exhibiting intense emotional labor (scores 51–70), though some also fell into the extremely low range (14–30). High resilience was significantly more common in the low fatigue group (93.39%) than in the high fatigue group (68.17%; *X* ^2^ = 60.31, *p* < 0.001). The most striking difference lay in job stress level (*X* ^2^ = 494.54, *p* < 0.001): 42.56% of the high fatigue group experienced high stress (scores 106–140), compared to a predominance of low stress in the low fatigue group, indicating that elevated job stress strongly associated with moderate or high change fatigue (Table 3).

**TABLE 3:** Univariate analysis of demographic characteristics across latent profiles of change fatigue among nurses (*N* = 1383).

Variables	Low change fatigue profile (*n* = 243)	Moderate change fatigue profile (*n* = 852)	High change fatigue profile (*n* = 289)	*x* ^2^ value	*p*
Gender				0.14	0.932
Male	17 (7.02)	66 (7.75)	22 (7.61)		
Female	225 (92.98)	786 (92.25)	267 (92.39)		
Age range				15.97	0.100
18–25 years old	19 (7.85)	61 (7.16)	16 (5.54)		
26–30 years old	61 (25.21)	255 (29.93)	81 (28.03)		
31–40 years old	118 (48.76)	422 (49.53)	162 (56.06)		
41–50 years old	35 (14.46)	92 (10.80)	27 (9.34)		
50–60 years old	8 (3.31)	22 (2.58)	3 (1.04)		
Above 60 years old	1 (0.41)	0 (0.00)	0 (0.00)		
Hospital grade				7.81	0.252
Grade A, Class III	187 (77.27)	666 (78.17)	227 (78.55)		
Grade B, Class III	44 (18.18)	120 (14.08)	35 (12.11)		
Grade A, Class II	10 (4.13)	60 (7.04)	25 (8.65)		
Others	1 (0.41)	6 (0.70)	2 (0.69)		
Marital status				5.53	0.237
Unmarried	64 (26.45)	231 (27.11)	63 (21.80)		
Married	173 (71.49)	589 (69.13)	218 (75.43)		
Divorced	5 (2.07)	32 (3.76)	8 (2.77)		
Professional title				8.48	0.205
Nurse	44 (18.18)	137 (16.08)	32 (11.07)		
Senior nurse	100 (41.32)	363 (42.61)	122 (42.21)		
Nurse‐in‐charge	85 (35.12)	307 (36.03)	123 (42.56)		
Associate chief nurse and above	13 (5.37)	45 (5.28)	12 (4.15)		
Educational background				4.44	0.618
Secondary technical school	1 (0.41)	4 (0.47)	0 (0.00)		
Associate degree	30 (12.40)	95 (11.15)	25 (8.65)		
Bachelor’s degree	207 (85.54)	737 (86.50)	256 (88.58)		
Postgraduate and above	4 (1.65)	16 (1.88)	8 (2.77)		
Work experience				12.30	0.265
Less than 1 year	8 (3.31)	17 (2.00)	5 (1.73)		
1–2 years	14 (5.79)	43 (5.05)	14 (4.84)		
2–5 years	37 (15.29)	158 (18.54)	46 (15.92)		
5–10 years	48 (19.83)	191 (22.42)	77 (26.64)		
10–20 years	103 (42.56)	351 (41.20)	127 (43.94)		
More than 20 years	32 (13.22)	92 (10.80)	20 (6.92)		
Work department	Low Change Fatigue Profile (*n* = 243)	Moderate Change Fatigue Profile (*n* = 852)	High Change Fatigue Profile (*n* = 289)	74.214	< 0.001
Surgery	33 (13.64)	128 (15.02)	37 (12.8%)		
Emergency department	15 (6.20)	122 (14.32)	38 (13.1%)		
Operating room	50 (20.66)	137 (16.08)	74 (25.6%)		
Intensive care unit	19 (7.85)	94 (11.03)	65 (22.5%)		
Internal medicine	70 (28.93)	204 (23.94)	47 (16.3%)		
Other	55 (22.73)	167 (19.60)	28 (9.7%)		
Employment type				1.31	0.52
Permanent position	52 (21.49)	186 (21.83)	54 (18.69)		
Contract‐based position	190 (78.51)	666 (78.17)	235 (81.31)		
Position				3.98	0.409
Nurse	219 (90.50)	777 (91.20)	265 (91.70)		
Head nurse or above	20 (8.26)	64 (7.51)	24 (8.30)		
Other	3 (1.24)	11 (1.29)	0 (0.00)		
Frequency of mid/night shifts per week				21.25	0.002
1‐2 times	138 (57.02)	469 (55.05)	141 (48.79)		
3‐4 times	32 (13.22)	147 (17.25)	68 (23.53)		
4 times or more	5 (2.07)	52 (6.10)	21 (7.27)		
0 times	67 (27.69)	184 (21.60)	59 (20.42)		
Daily overtime hours beyond standard 8‐hour workday				28.36	< 0.001
0–1 h	149 (61.57)	409 (48.00)	122 (42.21)		
Within 2 h	64 (26.45)	292 (34.27)	101 (34.95)		
2–5 h	25 (10.33)	133 (15.61)	51 (17.65)		
More than 5 h	4 (1.65)	18 (2.11)	15 (5.19)		
Daily sleep duration				46.66	< 0.001
Less than 4 h	1 (0.41)	6 (0.70)	7 (2.42)		
4–6 h	45 (18.60)	229 (26.88)	109 (37.72)		
6–8 h	177 (73.14)	593 (69.60)	167 (57.79)		
8 h or more	19 (7.85)	24 (2.82)	6 (2.08)		
Emotional labor level				118.55	< 0.001
Level 1 (14‐30)	0 (0.00)	1 (0.12)	8 (2.77)		
Level 2 (31‐50)	132 (54.55)	682 (80.05)	247 (85.47)		
Level 3 (51‐70)	110 (45.45)	169 (19.84)	34 (11.76)		
Psychological resilience level				60.306	< 0.001
Level 1 (25‐50)	2 (0.82)	2 (0.23)	3 (1.04)		
Level 2 (51‐75)	14 (5.79)	238 (27.93)	89 (30.80)		
Level 3 (76‐100)	226 (93.39)	612 (71.83)	197 (68.17)		
Work stress level				494.54	< 0.001
Level 1 (35‐70)	103 (42.56)	41 (4.81)	4 (1.38)		
Level 2 (71‐105)	134 (55.37)	722 (84.74)	162 (56.06)		
Level 3 (106‐140)	5 (2.07)	89 (10.45)	123 (42.56)		

### 3.4. Multivariate Logistic Regression Analysis of Latent Profiles of Change Fatigue

Multinomial logistic regression analysis, with the moderate fatigue profile as the reference category and applying Firth’s penalized likelihood method to address quasicomplete separation in emotional labor levels, revealed several significant predictors of profile membership (Table 4).

**TABLE 4:** Multinomial logistic regression analysis of factors associated with change fatigue profiles (*N* = 1383).

Variable	Low fatigue vs. moderate (reference)	*p*‐value	OR (95% CI)	High fatigue vs. moderate (reference)	*p* value	OR (95% CI)
	β (SE)			β (SE)		
*Department (surgery as reference)*						
Emergency department	0.210 (0.269)	0.438	1.234 (0.728, 2.123)	−0.281 (0.260)	0.285	0.755 (0.453, 1.266)
Operating theater	0.200 (0.281)	0.479	1.221 (0.703, 2.145)	−0.565 (0.290)	0.053	0.568 (0.318, 1.006)
Intensive care unit	0.203 (0.290)	0.488	1.225 (0.691, 2.192)	0.835 (0.255)	0.001	2.305 (1.401, 3.846)
Internal medicine	−0.296 (0.353)	0.408	0.744 (0.363, 1.491)	0.752 (0.266)	0.005	2.122 (1.261, 3.605)
Other departments	−0.484 (0.372)	0.195	0.617 (0.288, 1.275)	−0.172 (0.292)	0.559	0.842 (0.472, 1.497)

*Night shifts per week (0 as reference)*						
1‐2 times	−0.210 (0.247)	0.398	0.810 (0.489, 1.312)	0.425 (0.197)	0.034	1.530 (1.034, 2.251)
3‐4 times	−1.162 (0.517)	0.016	0.313 (0.100, 0.817)	0.165 (0.308)	0.599	1.179 (0.630, 2.136)
≥ 4 times	−0.015 (0.197)	0.938	0.985 (0.663, 1.451)	0.352 (0.195)	0.075	1.422 (0.965, 2.084)

*D* *a* *i* *l* *y* *o* *v* *e* *r* *t* *i* *m* *e* *h* *o* *u* *r* *s* (0–−1 *h* *a* *s* *r* *e* *f* *e* *r* *e* *n* *c* *e*)						
< 2 h	−0.274 (0.192)	0.155	0.760 (0.517, 1.109)	−0.229 (0.172)	0.183	0.795 (0.565, 1.114)
2–5 h	−0.466 (0.281)	0.095	0.627 (0.352, 1.082)	−0.309 (0.223)	0.165	0.734 (0.469, 1.134)
> 5 h	−0.007 (0.587)	0.991	0.993 (0.265, 3.958)	0.394 (0.416)	0.354	1.483 (0.637, 3.343)

*Sleep duration per day (<* *4* *h as reference)*						
4–6 h	0.423 (1.085)	0.724	1.527 (0.204, 24.690)	−0.528 (0.680)	0.457	0.590 (0.150, 2.449)
6–8 h	0.498 (1.076)	0.672	1.646 (0.225, 26.153)	−0.985 (0.675)	0.167	0.373 (0.096, 1.539)
≥ 8 h	1.482 (1.121)	0.187	4.402 (0.540, 73.706)	−1.107 (0.803)	0.182	0.330 (0.064, 1.691)

*Emotional labor level (14-30 points as reference)*						
31‐50 points (Level 2)	−0.597 (1.394)	0.713	0.550 (0.058, 21.645)	−1.954 (0.959)	0.03	0.142 (0.013, 0.834)
51‐70 points (Level 3)	0.022 (1.402)	0.988	1.023 (0.105, 30.715)	−2.360 (0.980)	0.01	0.094 (0.008, 0.582)

*Resilience level (25-50 points as reference)*						
51‐75 points	−1.589 (1.068)	0.156	0.204 (0.016, 1.857)	0.282 (0.996)	0.792	1.325 (0.117, 9.727)
76‐100 points	−0.171 (1.041)	0.879	0.843 (0.071, 7.299)	0.258 (0.995)	0.808	1.295 (0.115, 9.497)

*Job stress level (35-70 points as reference)*						
71‐105 points (Level 2)	−2.423 (0.215)	< 0.001	0.089 (0.058, 0.135)	1.670 (0.484)	< 0.001	5.310 (2.251, 16.327)
106‐140 points (Level 3)	−3.808 (0.460)	< 0.001	0.022 (0.008, 0.052)	3.487 (0.502)	< 0.001	32.706 (13.265, 103.366)
Constant	1.023 (1.926)	0.648	—	−0.904 (1.521)	0.585	—

*Note: β* = regression coefficient. Multinomial logistic regression with Firth’s penalized likelihood estimation was applied to address quasicomplete separation. Moderate fatigue group served as the reference category.

Abbreviations: CI = confidence interval, OR = odds ratio, SE = standard error.

Predictors of high fatigue profile:

Compared to the moderate fatigue group, nurses in the high fatigue group were significantly more likely to work in the ICU (OR = 2.31, 95% CI: 1.40–3.85, *p* = 0.001) and internal medicine (OR = 2.12, 95% CI: 1.26–3.61, *p* = 0.005). Operating theater showed a marginal trend toward reduced odds (OR = 0.57, 95% CI: 0.32–1.01, *p* = 0.053), though this did not reach conventional significance levels. Working 1–2 night shifts per week was associated with 53% increased odds of high fatigue membership compared to no night shifts (OR = 1.53, 95% CI: 1.03–2.27, *p* = 0.034).

Regarding emotional labor, compared with minimal emotional labor (Level 1, 14–30 points), both moderate (Level 2, 31–50 points: OR = 0.14, 95% CI: 0.01–0.83, p = 0.030), and high (Level 3, 51–70 points: OR = 0.09, 95% CI: 0.01–0.58, p = 0.010) levels were associated with significantly lower odds of high fatigue. This pattern suggests that minimal emotional labor engagement may represent a distinct risk configuration for high change fatigue, rather than indicating that high emotional labor is a risk factor.

Work‐related stress emerged as the strongest predictor: Level 3 stress (106–140 points) was associated with a 33‐fold increase in the odds of high fatigue (OR = 32.71, 95% CI: 13.27–103.37, *p* < 0.001) compared to Level 1 (35–70 points). Neither daily overtime hours nor sleep duration demonstrated statistically significant associations with high fatigue profile membership (all *p* > 0.05).

### 3.5. Predictors of Low Fatigue Profile

Conversely, nurses in the low fatigue group showed distinct protective patterns. Working 3–4 night shifts per week was associated with 69% lower odds of low fatigue membership compared to no night shifts (OR = 0.31, 95% CI: 0.10–0.82, *p* = 0.016), suggesting a nonlinear relationship where moderate night shift frequency may represent an adaptive pattern distinct from both absence and excess. Daily overtime of 2–5 h showed a marginal trend toward reduced odds of low fatigue (OR = 0.63, 95% CI: 0.35–1.08, *p* = 0.095).

Most notably, emotional labor Level 2 (31–50 points) showed a nonsignificant trend toward reduced odds of low fatigue compared to Level 1 (OR = 0.55, *p* = 0.713), while Level 3 showed essentially no difference (OR = 1.02, *p* = 0.988). This pattern, combined with the high fatigue group findings, suggests that minimal emotional labor engagement (Level 1) is associated with both increased odds of high fatigue and reduced odds of low fatigue, supporting the interpretation that deficient emotional labor may represent a distinct risk configuration. Work stress showed the strongest associations: Level 2 (OR = 0.09, *p* < 0.001) and Level 3 (OR = 0.02, *p* < 0.001) were both strongly negatively associated with low fatigue membership, underscoring low stress as a critical protective factor. Psychological resilience did not demonstrate statistically significant independent associations with low fatigue membership (Level 3: OR = 0.84, *p* = 0.879).

## 4. Discussion

### 4.1. Driving Mechanisms of Change Fatigue in the Southwestern China Context

This study reveals that change fatigue among nurses in Southwestern China is not a monolithic phenomenon but rather manifests as distinct risk profiles shaped by the intersection of workplace demands, individual resources, and regional contextual factors. Our findings demonstrate that 82.5% of nurses experience moderate to high levels of change fatigue, a prevalence that aligns with international estimates yet carries unique regional characteristics warranting nuanced interpretation.

### 4.2. Regional Specificity and Healthcare System Dynamics

Southwestern China’s distinctive healthcare landscape—characterized by uneven resource distribution, pronounced ethnic diversity, and accelerated reform implementation—creates conditions conducive to change fatigue development. Three interconnected contextual mechanisms appear to amplify fatigue burdens in this region.

1. Geographic and resource constraints: The mountainous topography of Southwestern China limits interhospital resource sharing and professional networking, isolating nurses in remote facilities and reducing access to peer support during organizational transitions. Unlike nurses in resource‐concentrated eastern coastal regions, Southwestern nurses often face simultaneous implementation of multiple reform initiatives—including tiered healthcare delivery system reform, electronic health record modernization, and nursing quality standardization—without adequate infrastructural support or phased implementation timelines. This “reform stacking” phenomenon may explain why our observed high‐fatigue prevalence (20.9%) approaches rates in high‐income countries despite substantially lower baseline resources.
2. Ethnic and cultural diversity: The region’s substantial ethnic minority populations require nurses to navigate culturally adapted care protocols alongside standard organizational changes, adding layers of complexity to role adaptation. Language barriers and differing health beliefs may amplify the cognitive load of change implementation, particularly when training materials and change communications are not culturally tailored. This cultural complexity may intensify the emotional labor demands already documented as a key fatigue predictor.
3. Healthcare reform intensity: As a focal region for national healthcare reform pilots, Southwestern China has experienced accelerated policy experimentation—including DRG/DIP payment reforms and nurse staffing ratio mandates—that often outpaces organizational capacity for absorptive change. The concentration of high‐fatigue nurses in ICU and internal medicine settings (48.1% combined) and the association with frequent night shifts (1–2/week) may reflect region‐specific staffing challenges and inadequate recovery time between reform implementation and clinical demands.

### 4.3. Comparative Context and Global Relevance

Prevalence estimates from North America, Europe, and Australia indicate that ≥ 60% of nurses experience clinically relevant levels of change fatigue, with rates following a “persistently high—slowly rising” trajectory [1, 2, 3, 6, 9]. Using a multicentre cross‐sectional sample of 1383 nurses, the present study is the first to apply LPA to this population in China. The distribution of fatigue profiles (high 20.9%, moderate 61.6%, and low 17.5%) closely mirrors findings from a U.S. survey of 6798 nurses across 29 states (high 22% and moderate 59%) [2], underscoring the global relevance of change fatigue as an occupational health hazard.

Multinomial logistic regression demonstrated that nurses with high‐level change fatigue were concentrated primarily in the ICU and internal medicine departments. This subgroup recorded the longest overtime hours, the shortest sleep duration, the highest emotional‐labor scores (85.47% scored 51–70), and the strongest perceived work stress (42.56% scored > 106). After adjustment for demographic covariates, these occupational factors remained independently associated with fatigue profile membership, indicating that modifiable work‐environment exposures—rather than stable individual traits—are the principal factors associated with change fatigue. The findings are consistent with evidence from high‐income countries and furnish an empirical basis for precision interventions targeted at high‐risk specialties.

### 4.4. Heterogeneity of Change Fatigue: Beyond a Single Continuum

The identification of three distinct fatigue profiles challenges the conventional conceptualization of change fatigue as a unidimensional construct [1, 17, 22]. Our person‐centered approach reveals that nurses experiencing change fatigue represent qualitatively different adaptation states rather than merely varying degrees of severity along a single continuum.

### 4.5. Profile‐Specific Characteristics and Clinical Implications

The predominant moderate‐fatigue class (61.6%), clustering around 3.8–4.9 on all six CFS items (“somewhat agree”), represents a critical “passive adaptation—resource depletion” limbo. This group’s intermediate positioning suggests eroding coping reserves that, without effective intervention, may rapidly progress to the high‐fatigue profile. This pattern mirrors observations during Australia’s national e‐health rollout, where initially moderate fatigue levels escalated to severe burnout within 12 months without organizational support [22]. The moderate‐fatigue group’s substantial size underscores the urgency of preventive interventions to halt progression toward high‐fatigue states.

In contrast, the low‐fatigue group (17.5%) demonstrates that resilience to change fatigue is achievable even within high‐pressure healthcare environments. Understanding the protective factors characterizing this group—including adequate sleep duration (≥ 6 h in > 80%), and manageable job stress levels—provides actionable targets for intervention design.

### 4.6. Theoretical Contribution to Occupational Health Research

Beyond applying LPA to a new population, this study advances the change fatigue literature by demonstrating three key theoretical propositions:

1. Qualitatively distinct adaptation states: Fatigue profiles are not merely ordinal severity levels but represent fundamentally different psychological configurations. The high‐fatigue profile reflects active exhaustion and disengagement, while the moderate‐fatigue profile represents a precarious equilibrium between adaptation and depletion. This distinction has critical implications for intervention matching.
2. Nonlinear mechanisms of emotional labor: Our findings challenge the assumption that emotional labor operates through simple dose–response relationships. Instead, both deficient engagement (Level 1) and excessive investment (Level 3) emerge as risk configurations. However, given the cross‐sectional design, we cannot determine whether this pattern reflects a true U‐shaped relationship or whether unmeasured confounders (e.g., emotional detachment and professional disengagement) explain the elevated risk among low scorers. This interpretation requires cautious framing and longitudinal validation.
3. Contextual dependence of resilience: Unlike previous studies demonstrating direct protective effects of resilience, our findings suggest that resilience may function as a context‐dependent resource whose benefits are mediated through reduced job stress and more adaptive emotional labor strategies, rather than exerting independent main effects on fatigue profiles.

These findings collectively support a configurational theory of occupational health, wherein risk and protective factors combine to produce emergent profiles that cannot be predicted from main effects alone. The entropy value of 0.95 indicates excellent classification accuracy, providing confidence that these profiles represent genuine subpopulations rather than statistical artifacts.

### 4.7. Implications for Precision Prevention

The heterogeneous structure supports Rudman’s contention that precision prevention must transcend one‐size‐fits‐all resilience training [17]. With an entropy of 0.95, our latent‐profile solution offers a robust platform for identifying and managing subpopulations. Staggered interventions—such as prioritizing flexible rosters for high‐fatigue nurses, resilience training for moderate‐fatigue nurses, and maintenance of protective factors for low‐fatigue nurses—may therefore be more cost‐effective than universal programs.

### 4.8. Emotional Labor and Change Fatigue: The Risk of Deficient Engagement

One of the most striking findings of this study is the pattern observed between emotional labor and change fatigue. Compared with minimal emotional labor (Level 1, 14‐30 points), both moderate (Level 2, 31‐50 points) and high (Level 3, 51‐70 points) emotional labor levels were associated with significantly lower odds of high fatigue membership (OR = 0.14 and OR = 0.09, respectively). This pattern suggests that deficient emotional labor engagement may represent a distinct risk configuration for high change fatigue, extending beyond the traditional linear resource‐depletion model.

### 4.9. Statistical Evidence for the Deficient Engagement Hypothesis.

The multinomial logistic regression results provide robust statistical support for this interpretation. The protective effects of moderate and high emotional labor relative to minimal engagement demonstrate that the lowest level of emotional labor—not the highest—is associated with the greatest risk of high fatigue membership. Conversely, for low fatigue membership, neither moderate (Level 2: OR = 0.55, 95% CI: 0.06–21.65, *p* = 0.713) nor high (Level 3: OR = 1.02, 95% CI: 0.11–30.72, *p* = 0.988) emotional labor levels showed significant associations. This pattern—where minimal emotional labor engagement (Level 1) is associated with both increased odds of high fatigue and reduced odds of low fatigue—suggests that deficient emotional labor may represent a distinct risk configuration associated with both high‐fatigue and reduced resilience profiles. Theoretical mechanisms: Drawing on Grandey′s emotion regulation theory and the broader emotional labor literature, we propose that nurses with minimal emotional labor engagement may exhibit emotional suppression or inadequate professional socialization [15, 23].

These individuals may lack the deep acting skills necessary to align internal feelings with display rules, resorting instead to maladaptive suppression strategies that create emotional dissonance and cognitive burden [16]. Alternatively, extremely low scores may reflect emotional detachment or cynicism—a form of disengagement that paradoxically increases vulnerability to change‐related stress by severing the protective connection between personal identity and professional role. This interpretation aligns with the “emotional exhaustion ⟶ depersonalization” progression described in burnout research, where emotional withdrawal represents a late‐stage coping failure rather than effective stress management. Our finding that Level 1 emotional labor predicts both high‐fatigue (OR reference) and reduced low‐fatigue membership supports this deficient engagement hypothesis. These nurses may represent a subgroup experiencing “quiet quitting”—present physically but disengaged emotionally—rendering them particularly vulnerable to change‐related stress.

### 4.10. Integration With Subscale Dynamics

Although we used the composite emotional labor score for profile classification, the observed pattern likely reflects heterogeneous subscale configurations. The high‐fatigue group with low emotional labor scores may exhibit low deep acting combined with moderate surface acting (indicating inadequate emotional investment), while the high‐fatigue group with high emotional labor scores may show high surface acting combined with high deep acting (indicating overinvestment and resource drain). This interpretation is supported by meta‐analytic evidence demonstrating that surface acting has stronger positive associations with emotional exhaustion (*r* = 0.42) than deep acting (*r* = 0.24) and that their interaction creates compounding effects on burnout [23]. Future research should examine these subscale‐specific patterns to refine intervention targeting.

### 4.11. Clinical Implications for Nursing Management

These findings suggest that intervention content should be tailored to nurses’ baseline emotional labor patterns: For nurses with minimal emotional labor engagement and high fatigue, interventions should focus on emotional skills training and professional identity strengthening to address emotional suppression and detachment. Strategies may include communication skills workshops emphasizing authentic emotional expression, mentorship programs pairing disengaged nurses with resilient colleagues, and reflective practice sessions to reconnect personal values with professional purpose.

For nurses with high emotional labor engagement and high fatigue, interventions should emphasize emotional boundary‐setting, mindfulness‐based stress reduction (MBSR), and reduction of display rule intensity to prevent resource depletion [24]. Strategies may include mindfulness‐based emotional regulation training, clearer organizational guidelines on emotional display expectations, and regular debriefing sessions to process emotionally challenging encounters.

This differentiated approach aligns with the EMOTION program findings, where mindfulness‐based emotional regulation training reduced surface acting by 23% and emotional exhaustion by 18% among healthcare workers [24], and extends these findings by suggesting that intervention matching should consider both the lower and upper ends of the emotional labor spectrum.

### 4.12. The Paradox of Resilience: Absence of Direct Effects and Implications for Multilevel Management

While bivariate analyses revealed that high resilience (CD‐RISC 76–100) was observed in 93.4% of the low‐fatigue profile compared to only 68.2% of the high‐fatigue profile, multinomial logistic regression failed to demonstrate statistically significant independent effects of resilience on profile membership (all *p* > 0.05). This paradox—where resilience appears protective in descriptive analyses but loses significance when controlling for workplace factors—suggests that resilience may operate primarily as a moderator rather than a main effect predictor, or that its benefits are mediated through reduced perceived job stress and more adaptive emotional labor strategies. This finding challenges simplistic “resilience training” approaches and supports the need for integrated organizational interventions that address structural stressors while building individual capacity.

### 4.13. Evidence Base for Resilience as a Mediated Resource

The gradient in resilience levels across fatigue profiles mirrors a Canadian longitudinal study in which every 10‐point increase in resilience reduced the risk of developing severe burnout over 12 months by 28% [25]. A nationwide Chinese survey further showed that resilience explained 53% of the variance in burnout after controlling for demographics (*β* = −0.53, *p* < 0.001) [26]. However, our null findings in multivariate models suggest that in the context of change fatigue specifically, resilience may not exert direct protective effects independent of workplace stress and emotional labor patterns.

Importantly, our data suggest that resilience may function as a threshold resource whose benefits require achievement of high levels (CD‐RISC ≥ 76) combined with supportive organizational contexts. The dramatic descriptive difference in high‐resilience prevalence between low‐ (93.4%) and high‐fatigue (68.2%) groups, coupled with null multivariate effects, implies that resilience‐building interventions will be most effective when implemented within contexts of manageable job stress and adaptive emotional labor regulation.

### 4.14. The Protective Matrix: Resilience and Organizational Support

Organizational and social support have also been shown to mediate the stress–fatigue–turnover intention pathway [27, 28]. High resilience coupled with strong organizational support, therefore, constitutes a critical protective matrix against change fatigue. This interaction suggests that resilience‐building interventions should be integrated with organizational support initiatives to maximize effectiveness—a finding consistent with the job demands‐resources (JD‐R) model’s emphasis on resource accumulation.

### 4.15. A Multilevel Management Framework

Management practice should shift from reactive remediation to proactive, multilevel design. We propose a comprehensive framework integrating individual, team, and organizational‐level interventions:

Individual‐level interventions: Resilience training programs targeting the CD‐RISC ≥ 76 threshold, sleep hygiene education given that 37.72% of high‐fatigue nurses slept < 6 h per night, and stress management workshops incorporating mindfulness techniques.

Team‐level interventions: Peer support networks for nurses in high‐risk specialties (ICU/internal medicine), regular team debriefing sessions to process change‐related stress, and mentorship programs pairing high‐resilience and high‐fatigue nurses.

Organizational‐level interventions: Integration of a “change‐fatigue index” into nursing‐quality sensitive indicators, workload redistribution in high‐risk specialties to reduce non‐clinical task burden, and flexible scheduling policies limiting consecutive night duties to ≤ 2 and total weekly overtime to ≤ 8 h [29].

The “Safer Nursing Care Tool,” validated in 17 European hospitals, provides a model for dynamically adjusting nurse staffing according to patient acuity [30]. Given that 37.72% of high‐fatigue nurses slept < 6 h per night, wearable sleep trackers could be linked to rostering systems to trigger pre‐emptive alerts when fatigue risk escalates.

### 4.16. Priority Setting for Resource Allocation

Our findings enable evidence‐based priority setting for intervention resource allocation:

Highest priority: Nurses in ICU/internal medicine with high work stress (Level 3) and suboptimal emotional labor regulation—this subgroup faces a 33‐fold increased risk of high fatigue and represents the most vulnerable population.

High priority: Nurses working 1–2 night shifts per week or > 2 h overtime daily—our workload analyses revealed increased high‐fatigue risk under these conditions, consistent with a 52‐study meta‐analysis showing that each additional overtime hour raises burnout risk among emergency nurses by 9% [31].

Moderate priority: Nurses with extreme emotional labor scores (either Level 1 or Level 3) who may benefit from targeted emotional regulation training.

Ultimately, resilience‐building measures should be embedded systematically—through flexible scheduling, supervisor coaching, and peer‐support networks—to create a multilayered defense architecture against change fatigue.

### 4.17. Limitations

Several limitations warrant consideration when interpreting these findings:

1. Cross‐sectional design: The cross‐sectional design precludes causal inference regarding the temporal sequence between workplace factors and change fatigue development. We cannot determine whether high job stress leads to change fatigue or whether fatigued nurses perceive greater stress. Future studies should employ ecological momentary assessment (EMA) to capture intraindividual fluctuations in fatigue and workload, or longitudinal designs to track profile transitions over time.
2. Sampling bias and generalizability: This study employed convenience sampling, which may introduce sampling bias by overrepresenting nurses with particular motivations or availability. The sample was predominantly from Grade A tertiary hospitals (78.09%), with limited representation from primary and secondary healthcare settings where change implementation dynamics and resource availability differ substantially. Additionally, the exclusion of nurses with postgraduate education and those on leave may have eliminated individuals with distinct stress experiences. These factors limit generalizability to the broader nursing workforce in Southwestern China and other regions with different healthcare system structures.
3. Common method bias: Reliance on self‐reported measures for all constructs (change fatigue, emotional labor, resilience, and job stress) may introduce common method bias, potentially inflating associations between variables. Although we used validated instruments with good psychometric properties and ensured anonymity to reduce social desirability bias, future research should incorporate multisource data (e.g., supervisor‐rated performance, objective HR data on night shifts and overtime, and physiological markers of stress) to enhance construct validity.
4. Measurement and classification: The categorization of continuous variables (emotional labor, resilience, and job stress) for multinomial logistic regression, while necessary to address separation issues and facilitate clinical interpretation, involves loss of information. Alternative approaches using continuous specifications with regularization techniques should be explored in future analyses.
5. Leadership inclusion: The inclusion of head nurses and supervisory staff (7.81% of the sample) may introduce response bias, as their leadership roles could influence their perception of organizational change. Although we attempted to minimize coercion through voluntary participation and anonymity, we cannot rule out the possibility that some participants felt implicit pressure to participate or respond favorably. Future studies should stratify analyses by position level or restrict samples to frontline staff only.

## 5. Implications for Nursing

Management: This study provides actionable, evidence‐based guidance for nursing leaders to mitigate change fatigue through precision prevention and targeted organizational interventions.

### 5.1. Profile‐Based Risk Stratification

The three‐profile structure (low 17.5%, moderate 61.6%, and high 20.9%; entropy = 0.95) enables leaders to move beyond universal approaches. We recommend integrating the 6‐item CFMS into routine occupational health surveillance, with quarterly screening to detect escalation from moderate to high fatigue. High‐fatigue nurses (20.9%) require immediate workload relief and mental health referral; moderate‐fatigue nurses (61.6%) represent a critical prevention window for resilience training and peer support; low‐fatigue nurses (17.5%) should serve as peer mentors and change champions.

### 5.2. Unit‐Level Workload Restructuring

The 2.31‐fold (ICU) and 2.12‐fold (internal medicine) increased odds of high fatigue demand urgent staffing analysis in these units. Leaders should limit consecutive night duties to ≤ 2 shifts, ensure ≥ 11 h rest between shifts, and enforce weekly overtime caps (≤ 8 h). The 53% increased risk with 1–2 night shifts weekly suggests that moderate, scheduled exposure with adequate recovery is preferable to erratic or excessive scheduling.

### 5.3. Differentiated Emotional Labor Training

The counterintuitive finding that minimal emotional labor engagement (Level 1) constitutes a distinct risk configuration—rather than high engagement—requires reconceptualization of emotional skills programs:

Low engagement (Level 1): Professional identity strengthening and authentic expression workshops to address emotional detachment or “quiet quitting.”

High engagement (Level 3): Boundary‐setting training and MBSR to prevent resource depletion, supported by evidence of 23% surface acting reduction and 18% emotional exhaustion reduction with MBSR [24].

### 5.4. Integrated Resilience Strategy

The null independent effect of resilience in multivariate models indicates that resilience‐building must be embedded within organizational support initiatives rather than implemented as standalone programs. Leaders should ensure manageable job stress and adaptive emotional labor regulation as prerequisites for resilience training effectiveness, targeting the CD‐RISC ≥ 76 threshold within supportive contexts.

### 5.5. Systemic Monitoring and Regional Adaptation

Leaders should institutionalize change fatigue assessment within nursing‐sensitive quality indicators, using unit‐level dashboards combining CFMS scores, overtime hours, and turnover rates. In ethnically diverse, resource‐constrained contexts such as Southwestern China, leaders must ensure culturally tailored change communications and phased implementation timelines to avoid “reform stacking.”

## 6. Conclusion

Change fatigue among Southwestern Chinese nurses exhibits a heterogeneous tripartite structure. Minimal emotional labor engagement (Level 1) was associated with higher odds of high fatigue compared with moderate and high levels, suggesting that deficient emotional labor may represent a distinct risk configuration. Both modifiable workplace factors (ICU/internal medicine placement, night shifts, job stress Level 3 OR = 32.71) and emotional labor patterns were associated with profile membership, supporting the potential value of organizational interventions and targeted emotional labor training. Region‐specific policies should account for Southwestern China’s reform intensity, resource constraints, and ethnic diversity. The application of Firth’s penalized likelihood to LPA‐derived profiles provides a methodological template for sparse data in nursing research.

### 6.1. Implications for Nursing Management Practice

Our findings carry immediate implications for nursing management in Southwestern China and comparable healthcare systems globally:

1. Profile‐based risk stratification: The three‐profile solution (entropy = 0.95) provides a robust framework for identifying nurses at different risk levels. We recommend integrating change fatigue assessment into routine occupational health surveillance, with differentiated intervention protocols for each profile.
2. Targeted interventions for high‐risk specialties: Nurses in ICU and internal medicine settings warrant priority attention, given their 2‐ to 2.3‐fold increased odds of high fatigue. Interventions should address modifiable workplace factors including overtime hours, night shift frequency, and nonclinical task burden.
3. Emotional labor regulation training: The finding that minimal emotional labor engagement is associated with increased odds of high fatigue suggests that intervention content should be tailored to nurses’ baseline emotional labor patterns. Nurses with minimal emotional labor engagement may benefit from emotional skills training and professional identity strengthening, while those with excessive engagement may require boundary‐setting training and MBSR.
4. Integrated resilience strategy: Given that resilience showed no independent protective effects in multivariate models, healthcare organizations should consider integrated interventions that combine resilience‐building with job stress reduction and emotional labor skills training. Such programs should target the CD‐RISC ≥ 76 threshold while ensuring supportive organizational contexts.
5. Nursing leadership action framework: As detailed in Section 6, nursing leaders should implement profile‐based surveillance, dual‐threshold emotional labor interventions, sleep protection policies, phased reform implementation, and leadership‐specific resilience programs to create multilevel defense architectures against change fatigue.

### 6.2. Directions for Future Research

Several avenues for future research emerge from our findings:

1. Longitudinal trajectory analysis: Our cross‐sectional design precludes causal inference regarding temporal sequences. Future studies should employ longitudinal designs to track profile transitions over time and identify predictors of progression from moderate to high fatigue.
2. Intervention effectiveness trials: The differentiated intervention strategies proposed based on our U‐shaped emotional labor findings require empirical validation through randomized controlled trials comparing tailored versus universal approaches.
3. Subscale‐level analysis: Future research should examine surface acting and deep acting subscales separately to refine understanding of the mechanisms underlying the composite emotional labor effects observed in this study.
4. Cross‐cultural validation: Replication studies in other regions of China and internationally would help determine whether the three‐profile structure and predictor effects generalize across healthcare system contexts.

### 6.3. Closing Remarks

Change fatigue among nurses in Southwestern China represents a significant occupational health concern with implications for workforce sustainability and patient care quality. Our findings demonstrate that this phenomenon is neither inevitable nor uniformly distributed—rather, it clusters into distinct risk profiles associated with modifiable workplace factors and buffered by context‐dependent personal resources. The identification of these profiles and their predictors provides an evidence‐based foundation for precision prevention approaches that can help safeguard nurses’ health while maintaining high‐quality patient care in an era of continuous healthcare transformation.

## Author Contributions

Lin Wang: conceptualization, methodology, data curation, and writing–original draft. Daoyuan Chai: conceptualization, methodology, and formal analysis. Mingyue Wu: investigation, data curation, and validation. Yi Tang: investigation and resources. Jie Mi: supervision, writing–review and editing, and project administration.

## Funding

This research received no specific grant from any funding agency in the public, commercial, or not‐for‐profit sectors.

## Conflicts of Interest

The authors declare no conflicts of interest.

## Data Availability

The data that support the findings of this study are available from the corresponding author upon reasonable request.
